# Hypertrophic pyloric stenosis following persistent pulmonary hypertension of the newborn: a case report and literature review

**DOI:** 10.1186/s12887-018-1270-0

**Published:** 2018-09-03

**Authors:** Shigeo Iijima, Daizo Ueno, Toru Baba, Akira Ohishi

**Affiliations:** Department of Pediatrics, Hamamatsu University School of Medicine, 1 – 20 – 1 Handayama, Hamamatsu, Shizuoka, 431 – 3192 Japan

**Keywords:** Arginine, Hypertrophic pyloric stenosis, Neonate, Nitric oxide, Persistent pulmonary hypertension of the newborn, Pulmonary hypertension

## Abstract

**Background:**

Although persistent pulmonary hypertension of the newborn (PPHN) and infantile hypertrophic pyloric stenosis (HPS) are both well-known diseases that occur in early infancy, PPHN complicated by HPS is rare. As nitric oxide (NO) is an important mediator of biological functions, on both the vascular endothelium and smooth muscle cells, the decreased production of NO might play a role in the pathogenesis of both PPHN and HPS. We present the case of a neonate who developed HPS following PPHN, including a detailed review on research published to date, and we discuss the pathogenesis of PPHN and HPS.

**Case presentation:**

A female neonate born at 38 weeks of gestation, weighing 3140 g, developed PPHN due to meconium aspiration syndrome. Intensive treatment with high frequency oscillations and inhaled NO were initiated, and sildenafil and bosentan were added. She gradually recovered. At 15 days of age, the patient developed recurrent vomiting after feeding and the diagnosis of HPS was made. Intravenous atropine therapy was started at 20 days of age, but the efficacy was clinically unsatisfactory. The coadministration with transdermal nitroglycerin improved the symptoms, and oral feeding was successfully re-introduced.

**Conclusions:**

Our patient recovered from both PPHN and HPS using NO-related medications. A decrease in NO synthesis is likely to be a common pathway for PPHN and HPS.

## Background

Persistent pulmonary hypertension of the newborn (PPHN) is a life-threatening syndrome of failed circulatory adaptation at birth with persistently increased pulmonary vascular resistance [1]. Inhaled nitric oxide (NO), which improves oxygenation through selective pulmonary vasodilation without causing systemic hypotension, is the mainstay of PPHN treatment. On the other hand, hypertrophic pyloric stenosis (HPS) is a common surgical cause of vomiting during early infancy [2]. This condition is characterized by an abnormal thickening of the pyloric sphincter muscle layer, creating an obstruction of the gastric outlet. Although the exact etiology and pathogenesis of HPS are unknown, one hypothesis is impaired function of acetylcholine and muscarinic receptors [3], and medical treatment with atropine, a cholinergic blocking agent with antimuscarinic activity, is an available alternative to pyloromyotomy [4–6]. Moreover, the increased sphincter tone is believed to be related to a decrease in NO production [7]. Recently in Japan, the efficacy of transdermal nitroglycerin, a NO donor, as a non-surgical treatment option was reported [8]. Although PPHN and HPS are well-known diseases, their co-occurrence is rare. In this report, we describe a neonate with PPHN who subsequently developed HPS and discuss the relationship between these two diseases.

## Case presentation

The patient was a female neonate born at 38 weeks of gestation, weighing 3140 g. She was delivered to a 34-year-old primigravida uncomplicated mother by emergency cesarean section due to fetal distress following idiopathic oligohydramnios which occurred during the third trimester. At birth, the infant was non-vigorous due to meconium aspiration and required resuscitation using endotracheal intubation and tracheal suctioning. The Apgar scores were 5 and 7 at 1 and 5 min, respectively. She required mechanical ventilation after transfer to the neonatal intensive care unit due to the development of respiratory insufficiency. A chest X-ray revealed bilateral diffused, grossly patchy opacities, which is a typical finding in meconium aspiration syndrome. Moreover, a > 10% difference between pre- and post-ductal saturation of peripheral oxygen, measured by pulse oximetry, persisted despite increased oxygen supplementation and ventilatory support. An echocardiogram revealed a structurally normal heart with marked tricuspid regurgitation, right-to-left shunting through the ductus arteriosus, and suprasystemic pulmonary pressures. Based on the clinical presentation and findings on imaging, a diagnosis of PPHN was made and intensive treatment with high frequency oscillations and inhaled NO were initiated. Subsequently, sildenafil, a specific phosphodiesterase type 5 inhibitor that enhances NO-mediated vasodilation, and bosentan, an endothelin receptor-inhibitor that increases internal activity of endogenous NO, were added to treatment. The symptoms gradually improved, and the infant was subsequently weaned from inhaled NO and ventilatory support, with extubation performed at 11 days of age.

Oral feeds with breast milk were started at 12 days of age. However, 3 days later, the infant developed non-bilious vomiting after feeding, which increased in frequency in the following days. The daily clinical examination was unremarkable and no mass was palpable in the abdomen. Plain abdominal X-ray at 17 and 19 days of age showed a dilated stomach. A contrast study was not performed. At 20 days of age, abdominal ultrasonography (US) revealed a hypertrophic pylorus, with a 5–6-mm mural thickening over a length of 18 mm, confirming the diagnosis of HPS. At this time, the plasma arginine level was 53.3 μmol/L (reference range: 53.6–133.6 μmol/L). The parents selected conservative treatment for their child, and atropine therapy was started. Atropine was administered intravenously at a dose of 0.1 mg/kg/day, which was divided equally by the number of oral feedings. Frequency of vomiting did not decrease in 3 days, and the dose was increased to 0.13 mg/kg/day. Subsequently, the frequency of vomiting decreased, but significant gastric residuals continued for more than 7 days after the start of atropine therapy. Hypertrophic pyloric muscle remained unchanged on repeated US. We considered the efficacy of the treatment was clinically unsatisfactory referring to previous studies [6, 9]. Then, transdermal nitroglycerin (5 mg/day) was added to the treatment at 29 days of age. Thereafter, the infant tolerated full enteral feeds, with no further vomiting, and oral feeding was successfully re-introduced. At 34 days of age, atropine was changed to oral administration at a dose of 0.2 mg/kg/day. Transdermal nitroglycerin was ceased over a day after that (the total dosing period was 7 days). Subsequently, the infant remained well and was discharged home at 43 days of age.

## Discussion

PPHN occurs in 2 per 1000 live births [1], with the incidence of HPS estimated at 1–8 in 1000 live births [2]. However, to the best of our knowledge, there are only five previously reported cases in the literature on the occurrence of both conditions in a patient [10–12]. The characteristics of those cases of HPS and PPHN or PH are summarized in Table 1 [10–13]. Brouwers et al. considered that if there was no common pathophysiologic pathway between HPS and PPHN, the combination of both conditions would occur in approximately 1 per 100,000 live births, and they proposed a deficiency in NO as a common pathway [10].

**Table 1:** Cases of hypertrophic pyloric stenosis following persistent pulmonary hypertension of the newborn or pulmonary hypertension

Study	Gestational age	Birth weight	Sex	PPHN or PH		HPS	
				Cause	Treatment	Age at onset	Treatment
Brouwers et al. [10]	39 weeks	2990 g	Male	MAS	ECMO	13 days	Pyloromyotomy
Malwade et al. [11]	Unknown	3200 g	Male	MAS	Sildenafil^a^	21 days	Pyloromyotomy
Present case	38 weeks	3140 g	Female	MAS	Inhaled NO^a^ Sildenafil^a^ Bosentan^a^	15 days	Atropine^a^ Nitroglycerin^a^
Robertson et al. [12]	37 weeks	3600 g	Male	CDH	Inhaled NO^a^ Epoprostenol Sildenafil^a^	40 days	Pyloromyotomy
Robertson et al. [12]	38 weeks	3714 g	Female	CDH	–	47 days	Pyloromyotomy
Robertson et al. [12]	38 weeks	2800 g	Male	CDH	ECMO Prostaglandin Milrinone	73 days	Pyloromyotomy
Ravindra et al. [13]	Term	2500 g	Male	CHD	–	2 months	Pyloromyotomy

*PPHN* persistent pulmonary hypertension of the newborn, *PH* pulmonary hypertension, *HPS* hypertrophic pyloric stenosis, *MAS* meconium aspiration syndrome, *CDH* congenital diaphragmatic hernia, *CHD* congenital heart disease, *ECMO* extracorporeal membrane oxygenation, *NO* nitric oxide

^a^ treatment associated with nitric oxide

**Fig. 1:**
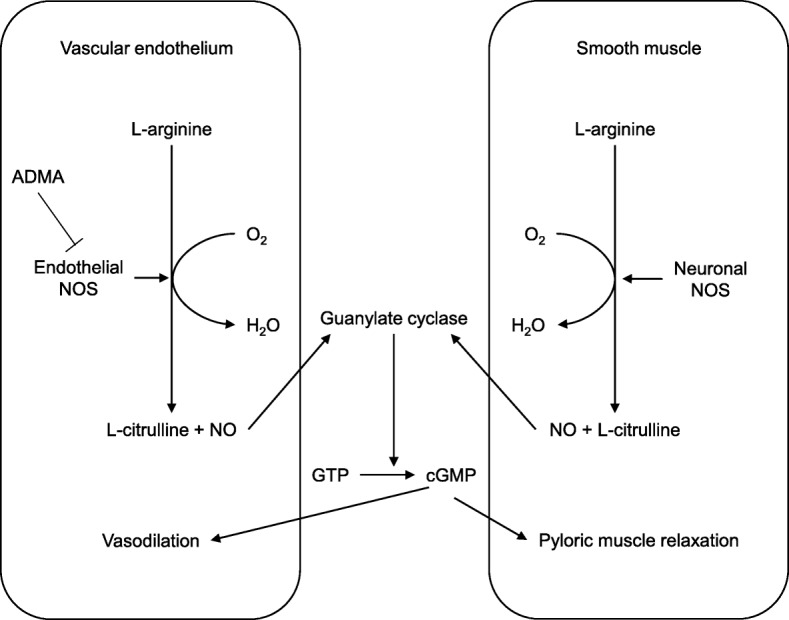
Diagram showing the mechanism of nitric oxide production that mediates vasodilation and smooth muscle relaxation. ADMA, asymmetric dimethylarginine; NOS, nitric oxide synthase; NO, nitric oxide; GTP, guanosine triphosphate; cGMP, cyclic guanosine monophosphate. ADMA is an inhibitor of endothelial NOS activity

NO has many physiological and pathological functions, and is endogenously synthesized from L-arginine by the family of NO synthetases (NOS) [14]. NOS is one of the most regulated enzymes in biology; in mammals, three isoforms have been identified, with two being constitutive (neuronal NOS [nNOS] and endothelial NOS [eNOS]) and the third being inducible NOS (Table 2) [14, 15]. NO is a potent vasodilator that is involved in the normal transition from fetal to neonatal circulation. In PPHN, an impairment in endothelial NO production was previously reported [1]. Hypoxia causing PPHN may reduce NO production through an uncoupling of NOS in pulmonary arterial endothelial cells [16]. On the other hand, NO has been demonstrated as a major inhibitory non-adrenergic and non-cholinergic neurotransmitter in the gastrointestinal tract, causing relaxation of the smooth muscle of the myenteric plexus upon its release [17]. There is evidence suggesting that impairment in NO synthesis contributes to HPS. Mice with reduced NOS activity developed grossly enlarged stomachs, with hypertrophy of the circular muscle wall [18]. In humans, decreases in serum NO and tissue expression of nNOS were observed in patients with HPS [7]. Therefore, NO deficiency may play a role in the pathogenesis of both PPHN and HPS. However, previous case reports have not demonstrated a substantial relationship between these two disorders and NO [10, 11]. In the case described by Brouwers et al., recovery from PPHN and HPS was obtained with treatments not associated with NO. Similarly, Malwade et al. did not provide a NO-related treatment for PPHN. In contrast, the present case of PPHN and HPS was successfully treated using inhaled NO, sildenafil, bosentan, and transdermal nitroglycerin. Regarding the HPS, transdermal nitroglycerin alone was not effective; coadministration with intravenous atropine was effective. Carr et al. demonstrated in their animal study that the inhibition of myopia by atropine is dependent on production of NO [19]. This suggests that the effect of atropine for HPS might also be mediated by NO and might support the efficacy of our treatment. Therefore, the approaches provided in our case all contributed to increasing NO in the affected organs. Regarding the mechanism of NO synthesis, eNOS, associated with PPHN, and nNOS, associated with HPS, do not have an association that can be explained by a single gene abnormality because these NOSs are encoded by different genes (Table 2) [14]. As NOS requires L-arginine for the production of NO regardless of the type, an impairment in arginine metabolism is likely to play an important role in NOS dysfunction. Therefore, the association between PPHN and HPS may be explained by a decrease in plasma concentration of arginine, leading to a deficiency in NO synthesis in the affected organ systems (Fig. 1). A previous study demonstrated that infants with PPHN had a low plasma concentration of arginine [20]. In HPS, Glass et al. reported an increase in plasma arginine levels in patients with HPS after surgery [21], but, to the best of our knowledge, there is no evidence of a decreased level of plasma arginine. In our patient, the plasma level of arginine at the time of diagnosis of HPS was borderline low, and we measured the arginine level only once for HPS, without measurement for PPHN. Further research is required to evaluate arginine metabolism in patients with either PPHN or HPS.

**Table 2:** Characteristics of different forms of nitric oxide synthase [14, 15]

Type	Gene(s)	Location	Function
Neuronal NOS (nNOS)	NOS 1 Chromosome 12 (12q24.22)	Nervous tissue Skeletal muscle type II	Cell communication: Neurotransmission Skeletal muscle contraction Sexual function Body fluid homeostasis
Inducible NOS (iNOS)	NOS 2 Chromosome 17 (17q11.2)	Immune system Cardiovascular system	Immune defense against pathogens: Inflammation Infection Malignant diseases Blood pressure regulation
Endothelial NOS (eNOS)	NOS 3 Chromosome 7 (7q36.1)	Endothelium	Cardiovascular homeostasis: Vasodilation Inhibition of platelet aggregation and adhesion Inhibition of vascular inflammation

*NOS* nitric oxide synthase

## Conclusions

The present patient recovered from both PPHN and HPS using NO-related medications. A decrease in NO synthesis is likely to be a common pathway for PPHN and HPS. If an infant recovering from PPHN develops recurrent vomiting, physicians should consider HPS as a complication.

## Abbreviations

CDH: Congenital diaphragmatic hernia
eNOS: Endothelial nitric oxide synthetases
HPS: Hypertrophic pyloric stenosis
nNOS: Neuronal nitric oxide synthetases
NO: Nitric oxide
NOS: Nitric oxide synthetases
PH: Pulmonary hypertension
PPHN: Persistent pulmonary hypertension of the newborn

## References

[1] Nair J, Lakshminrusimha S (2014). Update on PPHN: mechanisms and treatment. Semin Perinatol.

[2] Chung E (2008). Infantile hypertrophic pyloric stenosis: genes and environment. Arch Dis Child.

[3] Okazaki T, Yamataka A, Fujiwara T, Nishiye H, Fujimoto T, Miyano T (1994). Abnormal distribution of nerve terminals in infantile hypertrophic pyloric stenosis. J Pediatr Surg.

[4] Wu SF, Lin HY, Huang FK, Chen AC, Su BH, Li CI (2016). Efficacy of medical treatment for infantile hypertrophic pyloric stenosis: a meta-analysis. Pediatr Neonatol.

[5] Kawahara H, Imura K, Nishikawa M, Yagi M, Kubota A (2002). Intravenous atropine treatment in infantile hypertrophic pyloric stenosis. Arch Dis Child.

[6] Nagita A, Yamaguchi J, Amemoto K, Yoden A, Yamazaki T, Mino M (1996). Management and ultrasonographic appearance of infantile hypertrophic pyloric stenosis with intravenous atropine sulfate. J Pediatr Gastroenterol Nutr.

[7] Huang LT, Tiao MM, Lee SY, Hsieh CS, Lin JW (2006). Low plasma nitrite in infantile hypertrophic pyloric stenosis patients. Dig Dis Sci.

[8] Nagita A, Kosaka Y, Sakata R, Amemoto K, Okuda M, Ogita S (2006). Coadministration of transdermal nitroglycerin and intravenous atropine sulfate for hypertrophic pyloric stenosis. J Jpn Pediatr Soc.

[9] Meissner PE, Engelmann G, Troeger J, Linderkamp O, Nuetzenadel W (2006). Conservative treatment of infantile hypertrophic pyloric stenosis with intravenous atropine sulfate does not replace pyloromyotomy. Pediatr Surg Int.

[10] Brouwers AG, Waals-van de Wal CM (2009). Hypertrophic pyloric stenosis and pulmonary hypertension in a neonate. A common mechanism?. Acta Paediatr.

[11] Malwade S, Agarkhedkar S, Joshi H (2014). Persistent pulmonary hypertension and infantile hypertrophic pyloric stenosis in a neonate: reduced nitric oxide levels could be a common etiological factor. Med J DY Patil Univ.

[12] Robertson JO, Gadepalli SK (2017). Hypertrophic pyloric stenosis following repair of congenital diaphragmatic hernia. J Pediatr Surg Case Rep.

[13] Ravindra MN, Bhagya DV (2015). Dobutamine infusion for complex heart disease with pulmonary hypertension in an infant posted for open pyloromyotomy. Karnataka Anaesth J.

[14] Wang Y, Marsden PA (1995). Nitric oxide synthases: biochemical and molecular regulation. Curr Opin Nephrol Hypertens.

[15] Förstermann U, Sessa WC (2012). Nitric oxide synthases: regulation and function. Eur Heart J.

[16] Fike CD, Summar M, Aschner JL (2014). L-citrulline provides a novel strategy for treating chronic pulmonary hypertension in newborn infants. Acta Paediatr.

[17] Currò D, Ipavec V, Preziosi P (2008). Neurotransmitters of the non-adrenergic non-cholinergic relaxation of proximal stomach. Eur Rev Med Pharmacol Sci.

[18] Chung E, Curtis D, Chen G, Marsden PA, Twells R, Xu W (1996). Genetic evidence for the neuronal nitric oxide synthase gene (NOS1) as a susceptibility locus for infantile pyloric stenosis. Am J Hum Genet.

[19] Carr BJ, Stell WK (2016). Nitric oxide (NO) mediates the inhibition of form-deprivation myopia by atropine in chicks. Sci Rep.

[20] Vosatka RJ, Kashyap S, Trifiletti RR (1994). Arginine deficiency accompanies persistent pulmonary hypertension of the newborn. Biol Neonate.

[21] Glass RE, Goode AW, Houghton BJ, Rowell LW (1986). Plasma arginine in cancer of the gastrointestinal tract: effect of surgical treatment. Gut.

